# SGLT2 gene expression in thirst‐promoting NOS1 neurons and vasopressin‐synthesizing neurons in male rats

**DOI:** 10.14814/phy2.71100

**Published:** 2026-09-14

**Authors:** Takahiro Masuda, Masahide Yoshida, Young Chul Kim, Volker Vallon, Yoshiyuki Morishita, Daisuke Nagata, Tatsushi Onaka

**Affiliations:** ^1^ Division of Nephrology, Department of Internal Medicine Jichi Medical University Shimotsuke Tochigi Japan; ^2^ Division of Brain and Neurophysiology, Department of Physiology Jichi Medical University Shimotsuke Tochigi Japan; ^3^ Division of Nephrology and Hypertension, Department of Medicine and Pharmacology University of California San Diego and VA San Diego Healthcare System San Diego California USA

**Keywords:** fluid homeostasis, NOS1, oxytocin, sodium‐glucose cotransporter 2, thirst center, vasopressin

## Abstract

While sodium–glucose cotransporter 2 (SGLT2) inhibitors exert diuretic effects, they cause only mild changes in body fluid balance, partly through compensatory increases in water intake and vasopressin (AVP) secretion. However, whether SGLT2 inhibitors directly influence central pathways governing these fluid‐regulatory responses remains unclear. We here investigated *Sglt2* expression in thirst‐regulating centers, the organum vasculosum of the lamina terminalis (OVLT) and subfornical organ (SFO) and hypothalamic AVP‐synthesizing regions, including the supraoptic nucleus (SON) and paraventricular nucleus (PVN). Droplet digital PCR analysis revealed *Sglt2* expression in all regions, at levels comparable to angiotensin receptor 1a, a key regulator of drinking behavior. In situ hybridization confirmed *Sglt2* mRNA signals in OVLT, SFO, SON, and PVN. Double in situ hybridization revealed that *Sglt2* is co‐expressed in approximately 48%–60% of thirst‐promoting NOS1 neurons in the OVLT and SFO. Furthermore, *Sglt2* was detected in over 80% of AVP‐synthesizing neurons and approximately 60% of oxytocin neurons within the SON and PVN. Our findings suggest that SGLT2 inhibitors have the potential to directly modulate central thirst and neuroendocrine responses, offering a novel anatomical framework for understanding their systemic effects on fluid balance and cardio‐renal protection.

## 1 INTRODUCTION

The sodium‐glucose cotransporter 2 (SGLT2) is primarily expressed in the early proximal tubule of the kidney, where it plays a crucial role in glucose and sodium reabsorption (Rieg et al., 2014; Vallon, 2024; Vallon et al., 2011). By inhibiting SGLT2, a class of antihyperglycemic agents known as SGLT2 inhibitors reduces renal glucose reabsorption, leading to increased urinary glucose excretion and thus decreased blood glucose levels (Vallon, 2024). In addition, SGLT2 inhibitors exert multiple physiological influences, including natriuresis and osmotic diuresis (Masuda et al., 2020; Vallon, 2024). In clinical practice, SGLT2 inhibitors have beneficial effects on both cardiac and renal outcomes across diverse patient populations, including individuals with or without diabetes mellitus, chronic kidney disease (CKD) ranging from early to advanced stages, and various types of heart failure including individuals with reduced or preserved ejection fraction (Nuffield Department of Population Health Renal Studies G & SGLT2 inhibitor Meta‐Analysis Cardio‐Renal Trialists' Consortium, 2022). Consequently, these agents are increasingly employed to treat cardio‐renal diseases regardless of the presence or absence of diabetes, CKD, or heart failure (Masuda & Nagata, 2023; Nuffield Department of Population Health Renal Studies G & SGLT2 inhibitor Meta‐Analysis Cardio‐Renal Trialists' Consortium, 2022).

We previously reported that body fluid homeostasis is maintained in response to SGLT2 inhibition by stimulating water intake and vasopressin (AVP) secretion (Masuda et al., 2018; Masuda et al., 2020; Masuda et al., 2022; Masuda et al., 2024; Masuda & Nagata, 2023; Oka et al., 2023). AVP, also known as antidiuretic hormone, is crucial for water retention, primarily through regulating aquaporin‐2 expression in the renal collecting ducts (Christ‐Crain, 2019; Knepper et al., 2015; Masuda et al., 2020). Studies in both diabetic and non‐diabetic animal models suggest that SGLT2 inhibition stimulates water intake and AVP secretion as compensatory mechanisms to maintain body fluid balance (Masuda et al., 2018; Masuda et al., 2020; Masuda et al., 2022; Masuda et al., 2024).

The thirst center, primarily mediated by the organum vasculosum laminae terminalis (OVLT) and subfornical organ (SFO) (Bourque, 2008), responds to hyperosmolarity and hypovolemia, closely coordinating with AVP secretion to maintain systemic fluid homeostasis (Bourque, 2008). NOS1‐expressing neurons in the OVLT and SFO regulate drinking behavior in an activity‐dependent manner. These neurons also project to the hypothalamic supraoptic nucleus (SON) and paraventricular nucleus (PVN), which synthesize AVP (Augustine et al., 2018; Oka et al., 2015). AVP is synthesized in the SON and PVN, and its secretion is stimulated by increased serum/plasma osmolality, decreased blood volume, and various stressors (Kanbay et al., 2019; Knepper et al., 2015; Onaka, 2000). Interestingly, SGLT2 inhibitors have been shown to stimulate water intake and AVP secretion in human and animal studies in the absence of detectable increases in serum/plasma osmolality (Asakura‐Kinoshita et al., 2024; Eickhoff et al., 2019; Marton et al., 2024; Masuda et al., 2018; Masuda et al., 2020; Masuda et al., 2022; Masuda et al., 2024). Clinical studies have further confirmed that SGLT2 inhibitors increase serum levels of copeptin—a stable surrogate marker of AVP secretion (Knepper et al., 2015)—consistent with a role of the vasopressin system in the maintenance of body fluid volume during SGLT2 inhibition (Asakura‐Kinoshita et al., 2024; Bytyqi et al., 2025; Marton et al., 2024; Masuda, Asakura‐Kinoshita, et al., 2026; Nielsen et al., 2025; Oka et al., 2023; Scholtes et al., 2022). Facilitated AVP secretion can occur in response to SGLT2 inhibitors despite normal body fluid volume and normal osmotic conditions (Masuda et al., 2020; Masuda et al., 2022). It is, thus, possible that facilitated water intake and AVP secretion by SGLT2 inhibitors are not solely the consequence of changes in body fluid volume and osmolality. However, the mechanism by which SGLT2 inhibitors facilitate these responses remains unclear. In this regard, peripheral and central administration of SGLT2 inhibitors has been reported to alter neural activity in the hypothalamus (Nguyen et al., 2020; Takeda et al., 2021). Furthermore, SGLT2 inhibitors have been shown to cross the blood–brain barrier (Pawlos et al., 2021; Tahara et al., 2016), and SGLT2 immunoreactivity has been reported to exist in the brain (Chiba et al., 2020). Nevertheless, whether SGLT2 is actually expressed in thirst‐regulating centers and AVP‐synthesizing neurons remains largely unexplored.

In this study, we aimed to investigate the presence of SGLT2 in the circumventricular organs such as the OVLT and SFO and hypothalamus using molecular and histological approaches. By identifying *Sglt2* expression in specific functional neuronal populations, including *Nos1* and *Avp*‐expressing neurons, our findings provide an anatomical basis for direct central actions of SGLT2 inhibitors in modulating fluid homeostasis and neuroendocrine regulation.

## 2 MATERIALS AND METHODS

### 2.1 Animals

Male Sprague–Dawley rats (12–18 weeks old, CLEA Japan, Tokyo, Japan) were used in the present study. The rats were housed under a 12:12 h light/dark photocycle at 20°C–24°C and 40%–70% relative humidity. Food and water were available ad libitum. Rats were fed a standard laboratory rodent chow (CE‐2, CLEA Japan, Tokyo, Japan). All animal procedures were approved by the Institutional Animal Experiment Committee of Jichi Medical University (approval no. 24061‐01) and were conducted in accordance with the Institutional Regulations for Animal Experiments and Fundamental Guidelines for Proper Conduct of Animal Experiments and Related Activities in Academic Research Institutions under the jurisdiction of the Ministry of Education, Culture, Sports, Science and Technology.

### 2.2 Kidney and brain sampling

Rats were anesthetized with an i.p. injection of 200 mg/kg tribromoethanol (Avertin; Wako Pure Chemical Industries, Ltd., Osaka, Japan). Kidneys and brains were obtained after decapitation and immediately frozen in powdered dry ice and kept at −80°C until use.

### 2.3 RNA extraction and reverse transcription

Total RNA extraction and reverse transcription were conducted using a procedure reported previously (Masuda et al., 2024). Fresh frozen kidneys were cut coronally with a cryostat at a thickness of 30 μm. Kidney sections were stored in tubes. Fresh frozen brains were cut coronally with a cryostat at a thickness of 300 μm. Brain sections were mounted on glass slides. The brain tissues containing the OVLT, SFO, SON, and PVN were micro‐punched from the sections using Sample Corers (Fine Science Tools, Foster City, CA, USA). The diameter of the corer was 1.0 mm for the SFO, SON, and 2.0 mm for the OVLT and PVN. RNA treated with DNase I (#2270A, Takara Bio Inc., Shiga, Japan) was extracted from kidney tissues or micro‐punched brain tissues using RNeasy FFPE kit (#73504, Quiagen, Hilden, Germany). Extracted total RNA was reverse transcribed into first strand cDNA using a SuperScript IV First‐ Strand cDNA Synthesis System (#18091050, ThermoFisher scientific, Waltham, MA, USA).

### 2.4 Droplet digital PCR for transcripts quantification

Droplet digital PCR was conducted to measure the mRNA expression of *Sglt2* (official gene symbol: *Slc5a2*), *Agtr1a* (angiotensin II receptor, type 1a), and *Avp* using the QX200 Digital PCR System (Bio‐Rad Laboratories, Inc., California, USA) as previously reported (Masuda et al., 2024). *Polr2a* (RNA polymerase II subunit A), *Actb* (actin beta), *Tbp* (TATA‐box binding protein), and *G6pd* (glucose‐6‐phosphate dehydrogenase) were used as internal controls. Reaction mixture contained gene‐specific primers, hydrolysis probes, and ddPCR Supermix (#186‐3010, Bio‐RadLaboratories, Inc.). The gene‐specific primers and hydrolysis probes were shown in Table S1. The number of rats used was 4–5.

### 2.5 In situ hybridization

Fresh frozen brains were cut coronally with a cryostat at a thickness of 20 μm. All sections were mounted on silane‐coated glass slides and kept at −80°C until use. Every 8th section was then processed for mRNA detection as described previously (Usui et al., 2021). Digoxigenin (DIG)‐ or fluorescein‐labeled cRNA probes were used to detect single mRNA and multiple mRNAs simultaneously. cDNA fragments of rat SGLT2 (1–2254 bp; GenBank, U_29881), rat AVP (21–512 bp; GenBank, NM_016992), rat OXT (142–436 bp; GenBank, NM_012996), and rat NOS1 (99–1174 bp; GenBank, NM_052799) were subcloned into the pUC57 plasmid vector. In vitro transcription was performed using T3 RNA polymerase (#11031163001, Roche Diagnostics GmbH, Mannheim, Germany).

Sections were treated at room temperature with 4% paraformaldehyde in 0.1 M phosphate buffer (pH 7.4) for 15 min, 0.25% acetic anhydride in 0.1 M triethanolamine‐HCl (pH 8.0) for 10 min, and a hybridization buffer (50% formamide, 50 mM Tris–HCl, pH 7.5, 1 × Denhardt's solution, 0.6 M NaCl, 200 μg/mL yeast tRNA, 1 mM EDTA, and 10% dextran sulphate) for 30 min. Hybridization was performed at 63.5°C overnight in the hybridization buffer supplemented with cRNA probes at a dilution of 1:1000. Post‐hybridized sections were washed at 61°C with 5 × SSC containing 0.0005% Tween 20 for 30 min, 4 × SSC containing 50% formamide and 0.001% Tween 20 for 40 min, 2 × SSC containing 50% formamide and 0.001% Tween 20 for 40 min, and 0.1 × SSC containing 0.0005% Tween 20 for 30 min. Then the sections were incubated at room temperature in NTE buffer (0.5 M NaCl, 0.01 M Tris–HCl, pH 8.0, 5 mM EDTA, and 0.0005% Tween20) for 20 min, 20 mM iodoacetamide in NTE buffer for 20 min, and TNT buffer (0.15 M NaCl, 0.1 M Tris–HCl, pH 7.4, and 0.0005% Tween 20) for 10 min. The sections were then incubated at room temperature with DIG blocking solution (1% blocking reagent (#11096176001, Roche Diagnostics GmbH), 10% normal sheep serum (#S‐22, Merck Millipore, Darmstadt, Germany) in TNT buffer) for 30 min.

For chromogenic detection of the DIG‐labeled SGLT2 cRNA probe, sections were incubated at room temperature with alkaline phosphatase‐conjugated anti‐DIG antibody (dilution 1:500; Roche Diagnostics GmbH; RRID:AB_514497) for 90 min followed by visualization with NBT/BCIP solution (NBT 0.34 mg/mL, BCIP 0.18 mg/mL; #24720‐01 and #06278‐81, Nacalai tesque, Kyoto, Japan) in detection buffer (0.1 M Tris–HCl, pH 9.5, 0.1 M NaCl, and 50 mM MgCl_2_). The number of rats used was 4.

For double fluorescent detection, DIG‐labeled SGLT2 and fluorescein‐labeled NOS1 cRNA probes were detected by Cy5‐Tyramide Signal Amplification (TSA) and fluorescein‐TSA reagents, respectively. Fluorescein‐labeled AVP and OXT cRNA probes were directly detected without amplification. For detection of the DIG‐labeled SGLT2 cRNA probe, 0.5% TSA blocking reagent (#FP1020, PerkinElmer Life and Analytical Sciences, Boston, MA, USA) in TNT buffer for 30 min. Sections were incubated at room temperature with peroxidase‐conjugated anti‐DIG antibody (dilution 1:500; #11207733910, Roche Diagnostics GmbH; RRID:AB_514500) for 90 min followed by visualization with the Cy5‐TSA plus amplification kit (#NEL745001KT, PerkinElmer Life and Analytical Sciences). In *Nos1* and *Sglt2* double in situ hybridization experiments, the residual activities of the introduced peroxidase used to detect *Sglt2* mRNA were inactivated by incubating the sections in 1.0% H_2_O_2_ in TNT buffer at room temperature for 30 min. Then, the sections were processed to detect *Nos1* mRNA. The sections were incubated with a 1:500 dilution of a peroxidase‐conjugated anti‐fluorescein antibody (#11426346910, Roche Diagnostics GmbH; RRID:AB_840257) at room temperature for 90 min. Visualization was performed using the fluorescein‐TSA Plus amplification kit (#NEL741001KT, PerkinElmer Life and Analytical Sciences). Fluorescent images were acquired using a confocal laser scanning microscope (TCS SP5; Leica Microsystems, Wetzlar, Germany) equipped with a 40× PL APO oil‐immersion objective lens (NA 1.25, Leica Microsystems) and Leica Application Suite X software (Leica Microsystem). For each rat, the number of sections analyzed for double staining was four for the PVN and SON, and two for the OVLT and SFO. For the PVN and SON, positively stained cells were quantified bilaterally in each section. Total sample sizes were *n* = 4 rats for the PVN, SON, and SFO, and *n* = 3 rats for the OVLT.

## 3 RESULTS

### 3.1 SGLT2 was expressed in the thirst‐regulating regions and AVP‐expressing hypothalamic areas

**FIGURE 1:**
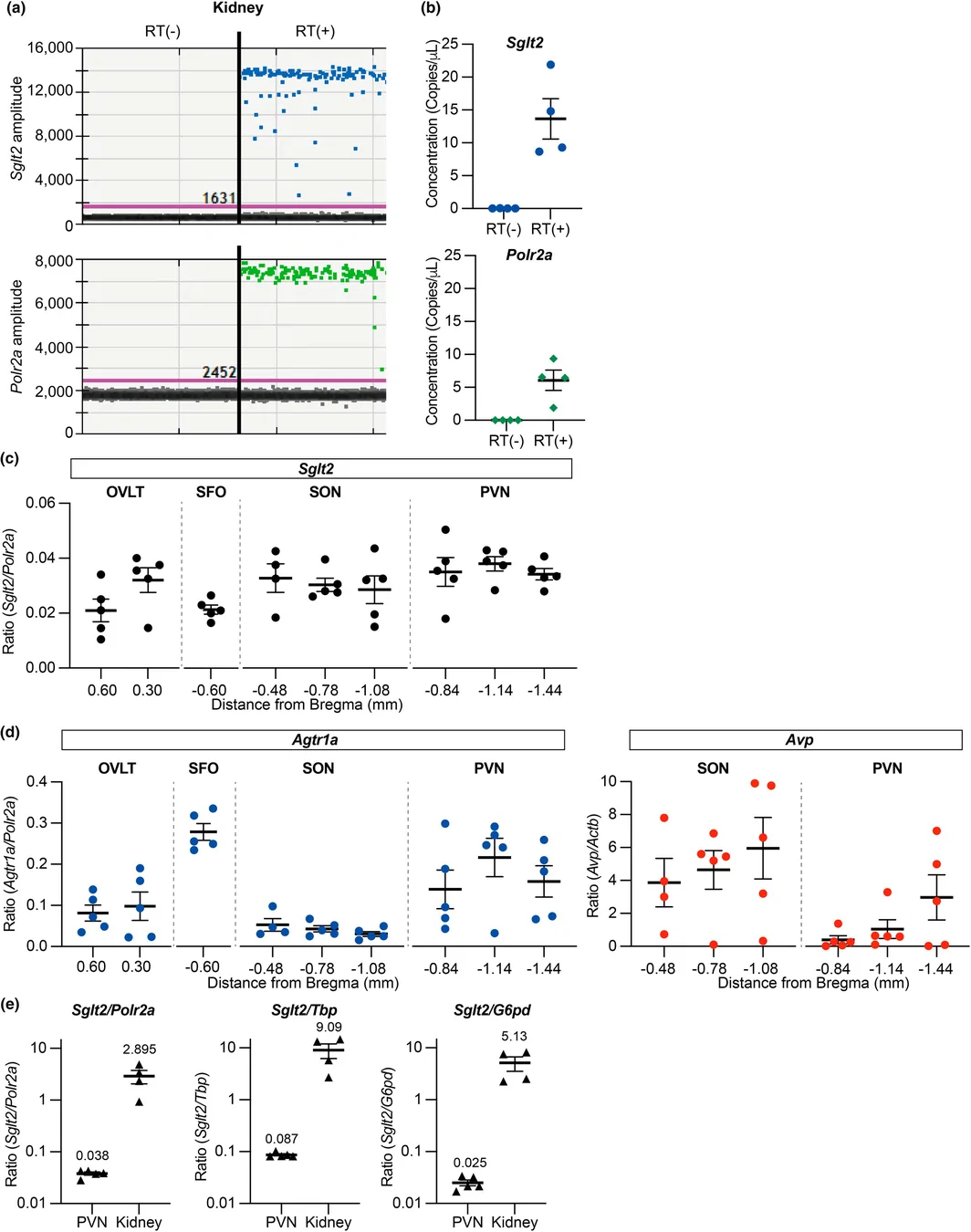
*Sglt2* gene expression analysis using droplet digital PCR in the kidney and brain. (a) Representative droplet plots of *Sglt2* (RTase(−)[upper left], RTase(+)[upper right]) and *Polr2a* (RTase(−)[lower left], RTase(+)[lower right]). (b) Transcript concentration of *Sglt2* (upper) and *Polr2a* (lower). The number of rats was 4. (c, d) Droplet digital PCR analysis for *Sglt2*, *Agtr1a*, and *Avp* transcript. The number of rats was 4–5. (e) Comparison of *Sglt2* expression in the PVN (bregma −1.44) and kidney using three different internal controls. The values in each column represent the mean. The number of rats was 4–5. Data are represented by mean ± SEM.

SGLT2 was expressed in the thirst center and in areas of AVP expression of the brain. We first used droplet digital PCR to analyze whether *Sglt2* is expressed in the OVLT, SFO, SON, and PVN. The quantification of *Sglt2* mRNA with droplet digital PCR was confirmed using mRNA from the kidney. Positive drops of RTase‐dependent *Sglt2* and *Polr2a* transcripts were detected (Figure 1a,b). *Sglt2* transcripts were detected in rat OVLT, SFO, SON, and PVN, and the *Sglt2*/*Polr2a* ratio was approximately 0.02–0.04 (Figure 1c). *Agtr1a*, which is known to be expressed in these four regions and regulates drinking behavior and AVP release, was also confirmed to be detected in these samples. The *Agtr1a*/*Polr2a* ratio was 0.03–0.27, i.e., similar to or only modestly higher than the ratios for *Sglt2*. In addition, *Avp* transcripts were also detected in the SON and PVN samples where *Sglt2* expression was detected (Figure 1d). Because the neuropeptide AVP is a highly expressed gene, *Actb*, which exhibits higher expression than *Polr2a*, was selected as an internal control. A comparison of expression levels with the kidney was carried out using the PVN sample with the highest *Sglt2* expression. Comparisons using three types of internal standard genes showed that *Sglt2* expression in the brain was 76–205 times lower than in the kidney.

**FIGURE 2:**
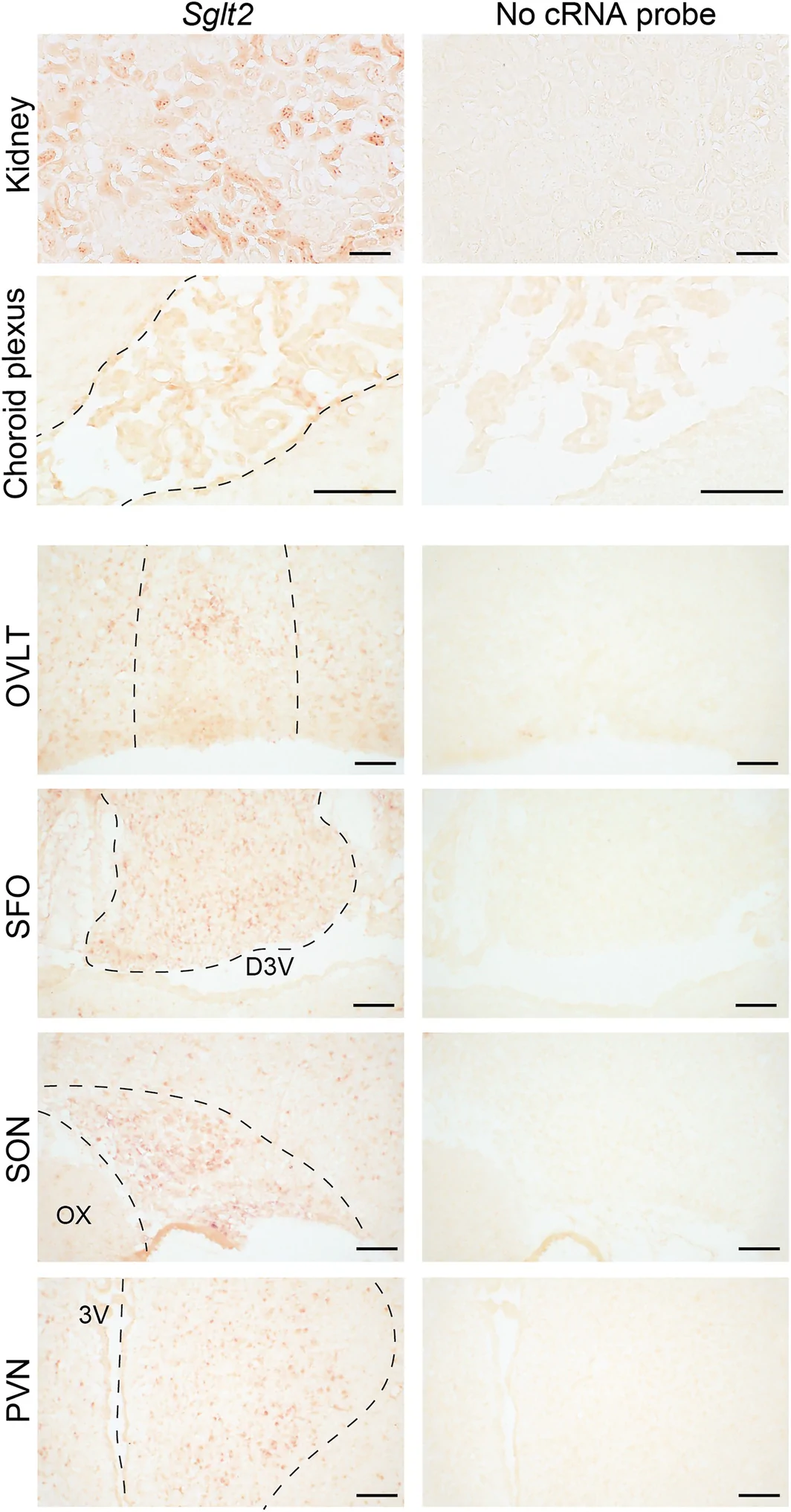
Expression of *Sglt2* mRNA in the kidney, choroid plexus, OVLT, SFO, PVN, and SON. Representative pictures of chromogenic in situ hybridization showing *Sglt2* mRNA. Negative control sections hybridized without cRNA probes (No cRNA probe; right panels). The number of rats was 4 for the kidney and 3 for the choroid plexus, OVLT, SFO, PVN, and SON. D3V, the dorsal third ventricle. 3 V, the third ventricle. OX, optic chiasm. Scale bars show 100 μm.

We next performed histological analysis of *Sglt2* expression in the OVLT, SFO, SON, and PVN of rats. *Sglt2* mRNA was detected in the proximal tubules of the kidney by in situ hybridization. In addition, positive *Sglt2* mRNA signals were observed in the choroid plexus. In the brain, as with droplet digital PCR, positive signals were detected in the OVLT, SFO, SON, and PVN (Figure 2).

### 3.2 *Sglt2* was expressed in NOS1 neurons

**FIGURE 3:**
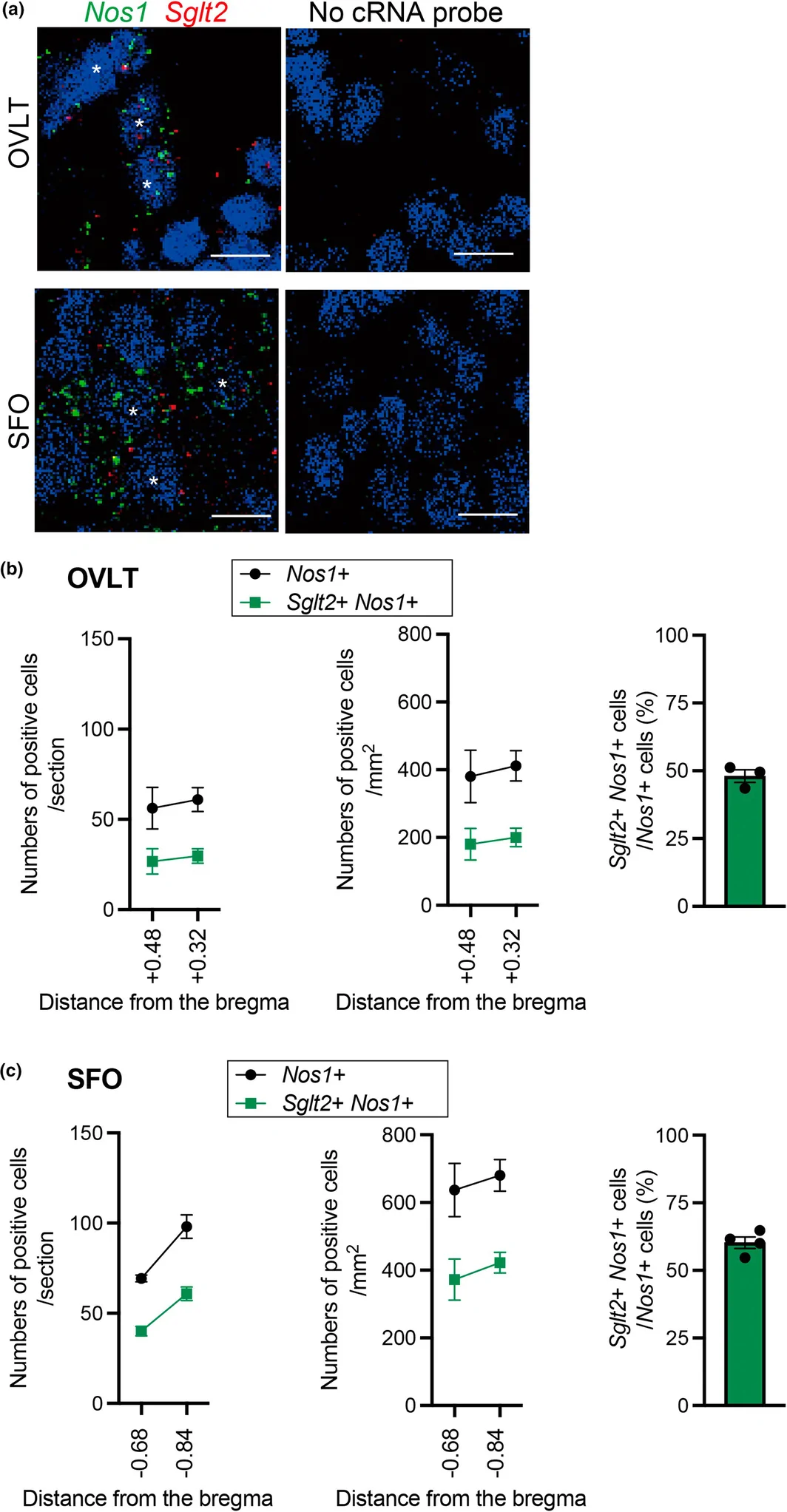
Expression of *Nos1* and *Sglt2* mRNA in the OVLT and SFO. (a) Double fluorescent in situ hybridization for *Nos1* mRNA (green dots), *Sglt2* mRNA (red dots) and nuclear with NucBlue (blue) in the OVLT and SFO. Negative control sections hybridized without cRNA probes (No cRNA probe; right panels). Asterisks represent double‐labeled neurons. (b) Quantitative analysis of *Nos1*‐positive (*Nos1*+) and *Sglt2*/*Nos1* double positive (*Sglt2*+ *Nos1*+) neurons in the OVLT, showing the numbers of positive cells per section (left) and per mm^2^ (middle) across different bregma coordinates. Percentage of *Sglt2*‐positive cells among *Nos1*‐expressing neurons in the OVLT (right). (c) Quantitative analysis of *Nos1*+ and *Sglt2*+ *Nos1*+ neurons in the SFO, showing the numbers of positive cells per section (left) and per mm^2^ (middle) across different bregma coordinates. Percentage of *Sglt2*‐positive cells among *Nos1*‐expressing neurons in the SFO (right). Scale bars show 10 μm. Data are represented by mean ± SEM.

To determine whether *Sglt2* is expressed in *Nos1*‐positive neurons, we performed double fluorescence in situ hybridization. In both the OVLT and SFO, *Sglt2* mRNA was clearly detected in neurons expressing the *Nos1* gene (Figure 3a). The specificity of the staining was confirmed by the absence of significant fluorescent signals in the negative control sections processed without cRNA probes (Figure 3a (right)). Quantification of cellular co‐localization revealed similar distribution patterns for both *Nos1*‐single‐positive and *Sglt2*/*Nos1*‐double‐positive cells (Figure 3b,c). Specifically, *Sglt2* mRNA was expressed in 48.1% of *Nos1*‐positive neurons in the OVLT (Figure 3b, right) and 60.3% in the SFO (Figure 3c, right).

### 3.3 *Sglt2* was expressed in AVP and OXT neurons

**FIGURE 4:**
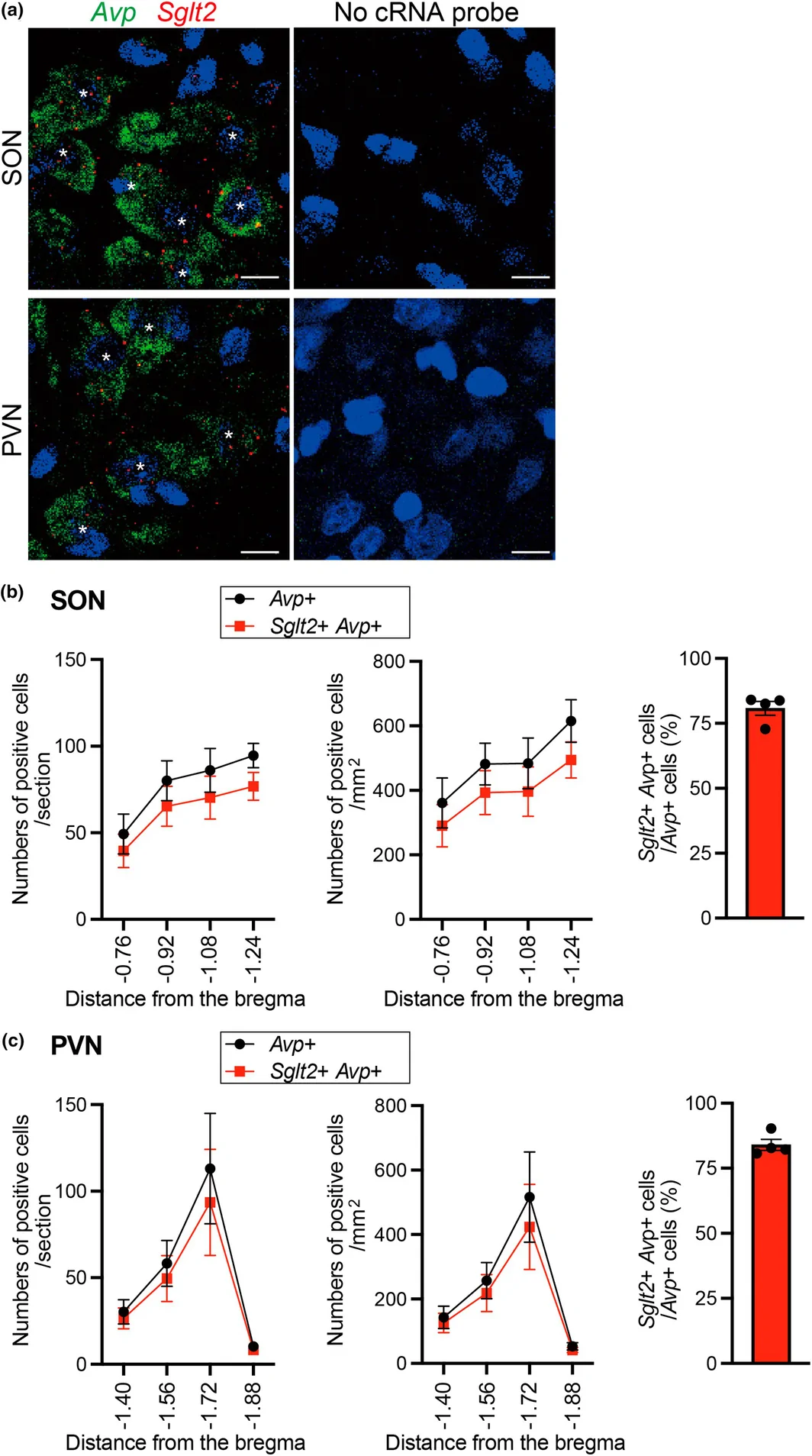
Expression of *Avp* and *Sglt2* mRNA in the SON and PVN. (a) Double fluorescent in situ hybridization for *Avp* mRNA (green), SGLT2 mRNA (red) and nuclear with NucBlue (blue) in the SON and PVN. Negative control sections hybridized without cRNA probes (No cRNA probe; right panels). Asterisks represent double‐labeled neurons. (b) Quantitative analysis of *Avp*‐positive (*Avp*+) and *Sglt2*/*Avp* double positive (*Sglt2*+ *Avp*+) neurons in the SON, showing the numbers of positive cells per section (left) and per mm^2^ (middle) across different bregma coordinates. Percentage of *Sglt2*‐positive cells among *Avp*‐expressing neurons in the SON (right). (c) Quantitative analysis of *Avp*+ and *Sglt2*+ *Avp*+ neurons in the PVN, showing the numbers of positive cells per section (left) and per mm^2^ (middle) across different bregma coordinates. Percentage of *Sglt2*‐positive cells among *Avp*‐expressing neurons in the PVN (right). Scale bars show 10 μm. Data are represented by mean ± SEM.

We analyzed whether *Sglt2* is expressed in *Avp*‐positive neurons using fluorescence double in situ hybridization. In both the SON and PVN, *Sglt2* mRNA was detected in neurons expressing the *Avp* gene (Figure 4a (left)). Quantitative analysis revealed that the distribution of *Avp*‐single‐positive cells and *Sglt2/Avp*‐double‐positive cells was similar across different anatomical levels (Figure 4b (left) and Figure 4c (middle)). In the SON, 80.8% of *Avp*‐positive neurons showed expression of *Sglt2* mRNA (Figure 4b (right)). In the PVN, 84.0% of *Avp*‐positive neurons showed expression of *Sglt2* mRNA (Figure 4c (right)).

**FIGURE 5:**
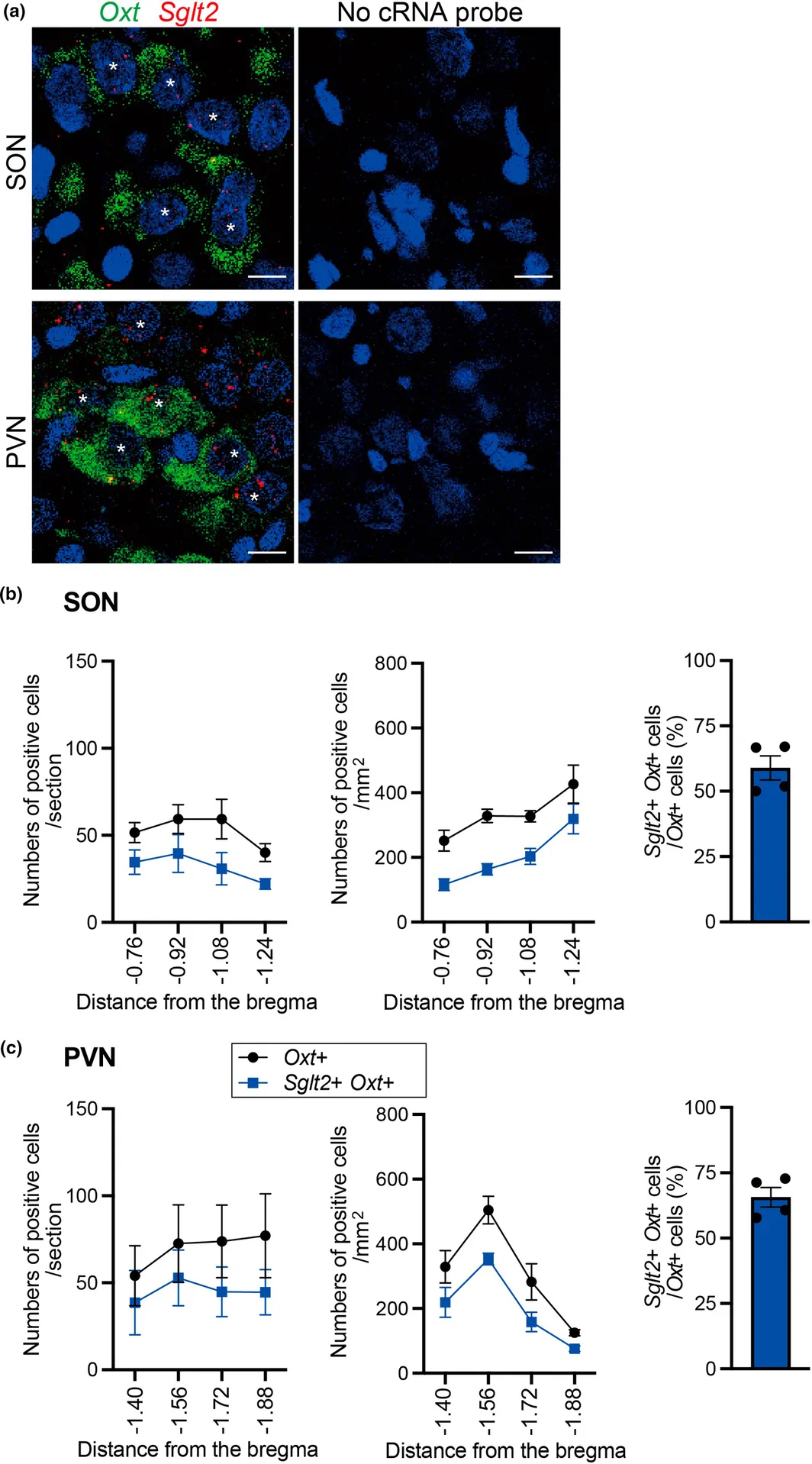
Expression of *Oxt* and *Sglt2* mRNA in the SON and PVN. (a) Double fluorescent in situ hybridization for *Oxt* mRNA (green), *Sglt2* mRNA (red dots) and nuclear with NucBlue (blue) in the SON and PVN. Negative control sections hybridized without cRNA probes (No cRNA probe; right panels). Asterisks represent double‐labeled neurons. (b) Quantitative analysis of *Oxt*‐positive (*Oxt*+) and *Sglt2*/*Oxt* double positive (*Sglt2*+ *Oxt*+) neurons in the SON, showing the numbers of positive cells per section (left) and per mm^2^ (middle) across different bregma coordinates. Percentage of *Sglt2*‐positive cells among *Oxt*‐expressing neurons in the SON (right). (c) Quantitative analysis of *Oxt*+ and *Sglt2*+ *Oxt*+ neurons in the PVN, showing the numbers of positive cells per section (left) and per mm^2^ (middle) across different bregma coordinates. Percentage of *Sglt2*‐positive cells among *Oxt*‐expressing neurons in the PVN (right). Scale bars show 10 μm. Data are represented by mean ± SEM.

Both the hypothalamic PVN and SON contain not only vasopressin neurons but also a distinct population of OXT neurons (Armstrong, 2015). We next examined whether *Sglt2* is expressed in OXT neurons. *Sglt2* mRNA was also found in neurons expressing the *Oxt* gene in the SON and PVN (Figure 5a). Quantitative analysis of the co‐localization of *Sglt2* and *Oxt* showed that 58.9% of SON *Oxt*‐positive neurons and 65.7% of PVN *Oxt*‐positive neurons expressed *Sglt2* mRNA (Figure 5b (right) and Figure 5c (right)).

## 4 DISCUSSION

SGLT2 inhibitors induce water drinking and AVP release, including in normovolemic and normoosmotic conditions (Masuda et al., 2020; Masuda et al., 2022). In this study, we discovered that *Sglt2* was expressed in thirst‐promoting NOS1 neurons as well as in AVP neurons. The co‐localization of *Sglt2* and *Nos1* was found to be approximately 50%–60%. The co‐localization of *Sglt2* and *Avp* was found to be 80% or higher. The present results suggest possible direct actions of SGLT2 inhibitors on the thirst center and AVP neurons to facilitate water intake and AVP secretion. These central effects may contribute to the broader fluid homeostasis–stabilizing actions of SGLT2 inhibitors that have been proposed to underlie their cardio‐renal benefits (Masuda, Kariro, & Morishita, 2026).

Recently, the central expression and functions of SGLT2 have gained increasing attention. While SGLT2 is well‐known for its renal actions, recent studies have revealed its presence in non‐neuronal brain elements: specifically, in choroid plexus epithelial cells and ependymal cells of both human and mouse brains (Chiba et al., 2020), as well as in brain pericytes, where it regulates glucose metabolism during ischemic stroke in non‐diabetic mice (Takashima et al., 2022). Consistent with these reports, we also detected *Sglt2* mRNA expression in the rat choroid plexus using in situ hybridization. In addition to non‐neuronal cells, SGLT2 and SGLT1 have been identified in neurons of the rostral ventrolateral medulla, a master regulator of sympathetic outflow. These findings suggest the existence of potential central mechanisms of SGLT inhibitors (Oshima et al., 2024). Extending these findings, the present study, utilizing cellular‐resolution analysis, provides the first direct evidence that SGLT2 is specifically expressed in key neurons controlling thirst regulation and AVP production.

Although SGLT2 is expressed at lower levels in key brain regions than in the kidney, it is present at levels comparable to those of other central regulators. In this study, local *Sglt2* expressions in the OVLT, SFO, SON, and PVN were 50–200 times lower than that of the kidney, the predominant expression site of SGLT2 in the body (Sabolic et al., 2012). Similarly, a recent mouse study proposed that SGLT2 protein levels in the choroid plexus were 4.5%–10.1% of those in the kidney of the same individual, though the specificity of the antibody used was not KO‐validated (Chiba et al., 2020). Notably, *Sglt2* mRNA was expressed in these brain samples at a level similar to that of *Agtr1a*, which is known to regulate drinking behavior in the brain (Mistlberger et al., 2001).

SGLT2 inhibitors can directly target thirst‐regulating brain regions to influence fluid homeostasis. SGLT2 inhibitors are well known to promote water intake (Masuda et al., 2018; Masuda et al., 2020; Masuda et al., 2022; Masuda & Nagata, 2023). In the present study, we identified *Sglt2* expression in thirst‐regulating centers. Importantly, the OVLT and SFO, which are key components of the t expressing hirst‐regulating system, are sensory circumventricular organs that lack a functional blood–brain barrier. Consequently, circulating SGLT2 inhibitors can directly reach these circumventricular organs. Notably, activation of NOS1 neurons in the OVLT and SFO drives drinking behavior (Augustine et al., 2018; Oka et al., 2015). Thus, SGLT2 inhibitors may exert their thirst‐inducing effects in part by enhancing the activity of these NOS1 neurons. Recent electrophysiological studies have demonstrated that NOS1 neurons in the SFO are excitatory thirst‐promoting neurons, in which membrane depolarization and inward currents increase neuronal excitability and drive drinking behavior (Zhang, Mak, et al., 2022). Therefore, the expression of *Sglt2* in these neurons provides an anatomical basis for the hypothesis that SGLT2 may directly influence the excitability of thirst‐promoting neurons, although this functional relationship remains to be established. We previously demonstrated that peripheral administration of ipragliflozin increased water intake in both diabetic and non‐diabetic rats (Masuda et al., 2018; Masuda et al., 2022; Masuda et al., 2024; Masuda & Nagata, 2020). Supporting a central mechanism and consistent with our findings, another study demonstrated that central administration of tofogliflozin similarly stimulated water intake in non‐diabetic rats (Takeda et al., 2021). Together, these findings provide an anatomical basis for a direct action of SGLT2 inhibitors on thirst‐regulating centers, although the precise mechanisms remain to be fully elucidated.

In the SFO, NOS1 neurons and glucose‐inhibited neurons share overlapping physiological characteristics, suggesting the possibility that a subset of NOS1 neurons functions as glucose sensors, integrating metabolic and thirst signals. Such coupled sensing of metabolic and fluid balance signals could be an evolutionarily conserved feature, echoing findings in the *Drosophila* brain (Jourjine et al., 2016). Glucose‐inhibited neurons, which are present in the hypothalamus and circumventricular organs, are cells that become excited in response to a decrease in intracellular glucose concentration (Burdakov & Gonzalez, 2009; Medeiros et al., 2012; Paes‐Leme et al., 2018). Electrophysiological studies have shown that approximately 27% of SFO neurons possess this glucose‐inhibited property. Crucially, the majority of these glucose‐inhibited neurons (approximately 71%) also respond to angiotensin II, a potent thirst‐inducing factor (Paes‐Leme et al., 2018). Moreover, NOS1 neurons in the SFO drive thirst and many express angiotensin II receptor 1 (Oka et al., 2015). These functional similarities support the notion that a specific subgroup of SFO NOS1 neurons functions as glucose‐inhibited neurons. This could position the NOS1/glucose‐inhibited neurons of the SFO as a neural hub that integrates systemic energy availability and fluid regulation signals to modulate drinking behavior. To speculate further, SGLT2 inhibitor‐induced reduction in intracellular glucose concentration may directly stimulate NOS1/glucose‐inhibited neurons in the SFO, triggering drinking behavior independent of the impending dehydration and fluid volume loss. Such a concept bears similarity with glucose sensing in renal macula densa cells, which share many functional characteristics of neurons (Gyarmati et al., 2024). In macula densa cells, SGLT1‐mediated glucose uptake enhances NOS1 activity to modulate tubuloglomerular feedback and increase glomerular filtration rate (Song et al., 2019; Zhang et al., 2019; Zhang, Cai, et al., 2022), thereby enhancing fluid excretion. In other words, SGLT‐dependent glucose sensing linked to NOS1 may favor a negative systemic fluid balance in both the kidney and the brain. Electrophysiological studies have demonstrated that SFO NOS1 neurons are excitatory thirst‐promoting neurons whose activation increases neuronal excitability and drives drinking behavior (Zhang, Mak, et al., 2022), but the functional interactions between glucose and NOS1 signaling and between the latter and neuron activity remain unknown. While our findings provide an anatomical foundation for this mechanism, direct functional validations—such as electrophysiological recordings or assessment of Fos expression in SGLT2‐expressing neurons—as well as characterizing how *Nos1* gene expression changes during SGLT2 inhibition, remain important subjects for future investigation.

SGLT2 inhibitors may modulate AVP secretion through both direct actions on AVP‐synthesizing neurons and indirect neural pathways via circumventricular organs. The expression of *Sglt2* in AVP‐synthesizing neurons of the SON and PVN, together with the ability of SGLT2 inhibitors to cross the blood–brain barrier (Pawlos et al., 2021; Tahara et al., 2016), provides a compelling basis for direct drug action. Previous reports showed increased AVP secretion during systemic SGLT2 inhibition (Masuda et al., 2020; Masuda et al., 2022; Masuda et al., 2024), but the effects of intracerebroventricular administration of SGLT2 inhibitors on AVP secretion have yet to be investigated and demonstrated. Furthermore, the expression of *Sglt2* in the OVLT and SFO suggests that these circumventricular organs are involved in the central response to SGLT2 inhibitors. Notably, NOS1 neurons in the OVLT and SFO are glutamatergic excitatory neurons that project directly to the SON and PVN, where AVP neurons are localized (Augustine et al., 2018). Therefore, SGLT2 inhibition in these sensory circumventricular organs has the potential to activate this excitatory projection, thereby facilitating downstream AVP secretion alongside established osmoregulatory pathways triggered by osmotic diuresis. Previous clinical studies demonstrated that copeptin levels remain strongly associated with plasma osmolality during SGLT2 inhibitor treatment (Berton et al., 2023; Oka et al., 2023), suggesting that osmotic regulation remains the primary determinant of AVP secretion. Therefore, while the present findings raise the possibility that SGLT2 signaling in the OVLT, SFO, SON, and PVN may modulate central fluid‐regulatory circuits, the relative contribution of direct central actions versus classical osmotic pathways remains unknown.

SON neurons express either AVP or OXT and project to the posterior pituitary. PVN AVP and OXT neurons include both magnocellular neurons projecting to the posterior pituitary and parvocellular neurons projecting to other brain areas (Armstrong, 2015). *Sglt2* was expressed not only in AVP neurons but also in SON and PVN OXT neurons. AVP and OXT play an important role in social behavior, stress‐related behavior, and memory learning (Perisic et al., 2024). It is tempting to speculate that SGLT2 inhibitors could also affect behaviors like these via AVP or OXT neurons in the brain.

A limitation of this study is that we did not directly assess the functional role of SGLT2 in the OVLT, SFO, SON, and PVN. To clarify whether central SGLT2 inhibition affects drinking behavior and AVP secretion, further studies using intracerebroventricular administration or SGLT2 knockdown models are warranted. Global SGLT2 knockout mice exhibit increased water intake and AVP secretion compared to wild‐type mice (Vallon et al., 2011). However, given that drinking behavior and AVP secretion are strongly influenced by body fluid homeostasis, it will be particularly necessary to use brain region‐specific SGLT2 knockout animals to elucidate the precise role of SGLT2 in the brain.

In conclusion, the present study provides the first cellular‐resolution evidence that SGLT2 is specifically expressed in key fluid‐regulatory neurons, including thirst‐promoting NOS1 neurons in the OVLT and SFO, as well as AVP‐synthesizing neurons in the SON and PVN. Our findings establish a novel anatomical framework in which SGLT2 inhibitors can modulate central dipsogenic and neuroendocrine responses by acting directly in the brain. By linking potential central metabolic sensing to fluid‐regulatory circuits, these proposed mechanisms provide new insights into how SGLT2 inhibitors affect systemic fluid homeostasis. Defining these key SGLT2‐mediated pathways will enhance our understanding of the brain–kidney axis and clarify the neural contributions to the extensive cardio‐renal benefits of SGLT2 inhibitors.

## AUTHOR CONTRIBUTIONS

**Takahiro Masuda:** Conceptualization; data curation; formal analysis; funding acquisition; investigation; methodology; project administration; validation. **Masahide Yoshida:** Conceptualization; data curation; formal analysis; funding acquisition; investigation; methodology; validation. **Young Chul Kim:** Methodology; validation. **Volker Vallon:** Funding acquisition; supervision; validation. **Yoshiyuki Morishita:** Validation. **Daisuke Nagata:** Funding acquisition; validation. **Tatsushi Onaka:** Conceptualization; funding acquisition; investigation; methodology; supervision; validation.

## FUNDING INFORMATION

This work was supported in part by KAKENHI grants from the Japan Society for the Promotion of Science (grant no. 15K21321 and 25K11504 [to T.M.]; 20K07264 and 24K10028 [to M.Y.]; and 24K02863 [to T.O.]), AMED‐CREST (grant no. JP23gm1510012 [to M.Y.]), the Manpei Suzuki Diabetes Foundation Number: 32436025 [to T.M.], Salt Science Research Foundation Number: 2232 [to T.M.], and by National Institutes of Health (NIH) Grant R01 DK112042 [to V.V.].

## CONFLICT OF INTEREST STATEMENT

Over the past 24 months, V. Vallon has served as a consultant and received honoraria from Astra‐Zeneca and Boehringer Ingelheim and received grant support for investigator‐initiated research from Boehringer Ingelheim, Gilead, Lexicon, Maze Therapeutics, and Novo‐Nordisk. All other authors declare that there is no conflict of interest regarding the present study.

## Supporting information


Data S1.

## Data Availability

The datasets analyzed during the current study are available from the corresponding author on reasonable request.
